# Supportive periodontal therapy and periodontal biotype as prognostic factors in implants placed in patients with a history of periodontitis

**DOI:** 10.4317/medoral.19136

**Published:** 2013-05-31

**Authors:** Luis A. Aguirre-Zorzano, Francisco J. Vallejo-Aisa, Ruth Estefanía-Fresco

**Affiliations:** 1 Professor of Periodontics (UPV/ EHU); 2 Postgraduate Periodontics course (UPV/ EHU)

## Abstract

Objectives: To evaluate bone loss around implants placed in patients with a history of treated chronic periodontitis and who did or did not attend supportive periodontal therapy, after one year in function. Furthermore, the influence of periodontal biotype and level of plaque was also evaluated. Material and Methods: Forty-nine patients participated voluntarily in the study. All subjects had a history of chronic periodontitis, which had been previously treated. After the active treatment, 27 patients attended supportive periodontal therapy (SPT) and the rest did not (No SPT). The O’Leary plaque index and periodontal biotype were recorded for each subject and 246 Astra Tech® OsseospeedTM implants were radiographically analysed (123 placed in SPT patients and 123 in No SPT patients) at the time of loading and one year later, measuring marginal bone loss with the program Dental Studio NX 6.0®. The statistical analysis was performed with Windows SPSS, applying Pearson’s correlation index and the Kruskal-Wallis and U-Mann Whitney non-parametric tests. Results: Six patients were found to have periimplantitis and sixteen mucositis. The survival rate was 99.59% (100% SPT and 99.18% No SPT). Mean bone loss was 0.39 mm (range [-0.71 - 8.05]). Among SPT patients, 95% of the implants had losses less than or equal to the mean (mean bone loss of 0.16 mm) compared to 53.7% for the No SPT group (mean bone loss of 0.62 mm). A statistically significant relationship was demonstrated between bone loss around the implant and the patient’s periodontal biotype and plaque index. Conclusions: The marginal bone loss around implants in patients with treated chronic periodontitis is minimal if they are in a controlled SPT programme and there is individual control of plaque index. Moreover, the presence of a thin periodontal biotype represents a risk factor for additional bone loss.

** Key words:**Peri-implantitis, chronic periodontitis, bacterial plaque, periodontal biotype.

## Introduction

Dental implants have become predictable in the treatment of totally or partially edentulous patients (1,2). The survival of implants is high, but not free of complications, being one of the most frequent periimplant inflammation (3). We refer to two conditions when discussing this disease: periimplant mucositis and periimplantitis. Mucositis is defined as an inflammatory lesion of infectious origin residing in the periimplant mucosa; when this involves the loss of supporting bone it would be referred to as periimplantitis. The presence of reddening, inflammation and bleeding on probing would assist in the diagnosis of the first entity and, if an increase in probing depth and suppuration together with marginal supporting bone loss, confirmed by X-ray, is also present, it would indicate periimplantitis (4,5).

 The infectious ethiology of periimplantitis has been confirmed in several papers (3,6-9). Similarly, bone loss has been linked to the presence of risk factors such as systemic diseases (10), the type of implant surface (11) and smoking (12) among others.

The periodontal biotype concept was established by Seibert and Lindhe in 1989 (13). Claffey and Shanley (14) proposed a thickness lower than 1.5 millimetres for the mucosa for thin biotypes and greater than or equal to 2 millimetres for thick biotypes, and suggested that the former are more susceptible to plaque accumulation and pathologies (inflammation, bleeding and recession). Furthermore, the importance of having good keratinised mucosa around implants in order to achieve predictable aesthetic results is well known (15,16).

The objective of this study was to evaluate bone loss around Astra Tech® implants placed in patients with a history of treated chronic periodontitis who did or did not attend supportive periodontal therapy, after one year in function, and the influence that periodontal biotype and level of plaque may have.

## Material and Methods

Forty-nine subjects, 18 men and 31 women, with a mean age of 47.6 years [38-66], who had been treated in the Postgraduate Periodontics Department of Universidad del País Vasco/Euskal Herriko Unibertsitatea for chronic periodontitis and who subsequently received oral rehabilitation with implant-supported prosthesis, were scheduled for reevaluation after having the prosthesis in function for one year. Despite being informed of the importance of receiving supportive periodontal therapy (SPT), 27 attended the appointments (SPT) and 22 did not (No SPT). None of these patients were suffering from systemic diseases, five presented craneo-mandibular dysfunction and only three were occasional smokers.

All data were collected after the first year in function and the patients participated voluntarily in the study, after being properly informed.

A 4-monthly visit protocol was established, which included: a) evaluation of plaque index (17), b) determination of probing depth and gingival index (18) by means of a periodontal probe (Williams Hu-Friedy, Chicago, IL, USA); c) professional prophylaxis in implants (titanium curettes, carbon-fibre curettes, rubber cup and prophylaxis paste), and teeth (piezoelectric ultrasound with a frequency of 30 KHz [H3, Suprasson®, Satelec, Bordeaux, France] and Gracey Curettes [Hu-Friedy, Chicago, IL, USA]), d) occlusal analysis with 12 micron articulating paper (Bausch Arti-Fol® Metallic BK28; KG, Cologne, Germany), e) X-rays (orthopantomography or intraoral X-rays performed using the parallel technique) every 6 months and f) reinforcement of personal motivation to control plaque.

A total of 246 Astra Tech® Osseospeed™ implants were analysed (Astra Tech AB, Mölndal, Sweden), along with their prostheses: 84 fixed metal-ceramic prostheses (single-unit and bridges), 14 fixed metal-resin hybrid prostheses and one over-denture. All of these prostheses substituted teeth that were lost as a result of the periodontal disease suffered.

Periodontal biotype was evaluated according to the anatomical characteristics of each patient and according to two standards: thin biotype or thick biotype (19).

**Figure 1:**
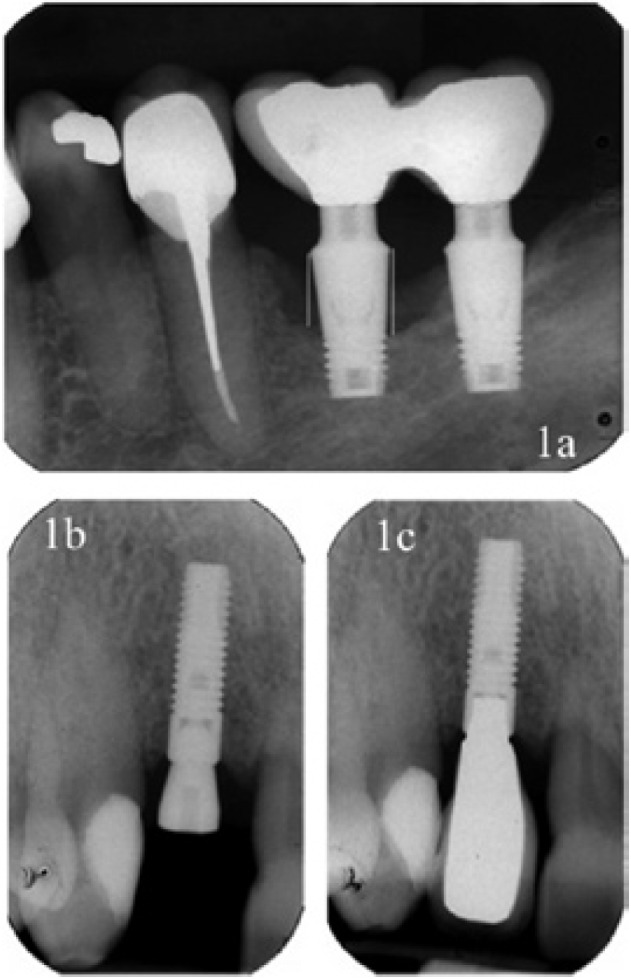
a) X-ray containing the references used in the measurements made from the implant shoulder to bone level, both mesially and distally. b) X-ray at the time of implant loading, showing that the bone margin coincides with the implant shoulder. c) X-ray after one year of functional loading in a patient who has received supportive periodontal therapy. The maintenance of the bone level can be observed.

Bone loss was measured from the implant abutment to the most coronal bone-to-implant contact point, both mesially and distally (Fig. 1) with the programme Dental Studio NX version 6.0® (NEMOTEC) over pre-scanned orthopantomography and intraoral X-rays at the time of loading (Fig. 1) and one year after (Fig. 1).

The statistical analysis of the data was performed with the Windows software SPSS version 14.0 and was based on the analysis of mean bone loss. Descriptive analyses of means, correlations and non-parametric contrasts were performed with the aim of finding significant differences between the groups defined by periodontal biotype and those who received or did not receive supportive periodontal therapy.

The failure of the data to confirm the normality hypothesis meant that ANOVA or t-tests could not be performed; the non-parametric tests, U-Mann Whitney to contrast two samples, and Kruskal-Wallis to contrast more than two samples were therefore performed. As the plaque index is a continuous quantitative variable, Pearson’s Correlations were performed.

## Results

**Figure 2:**
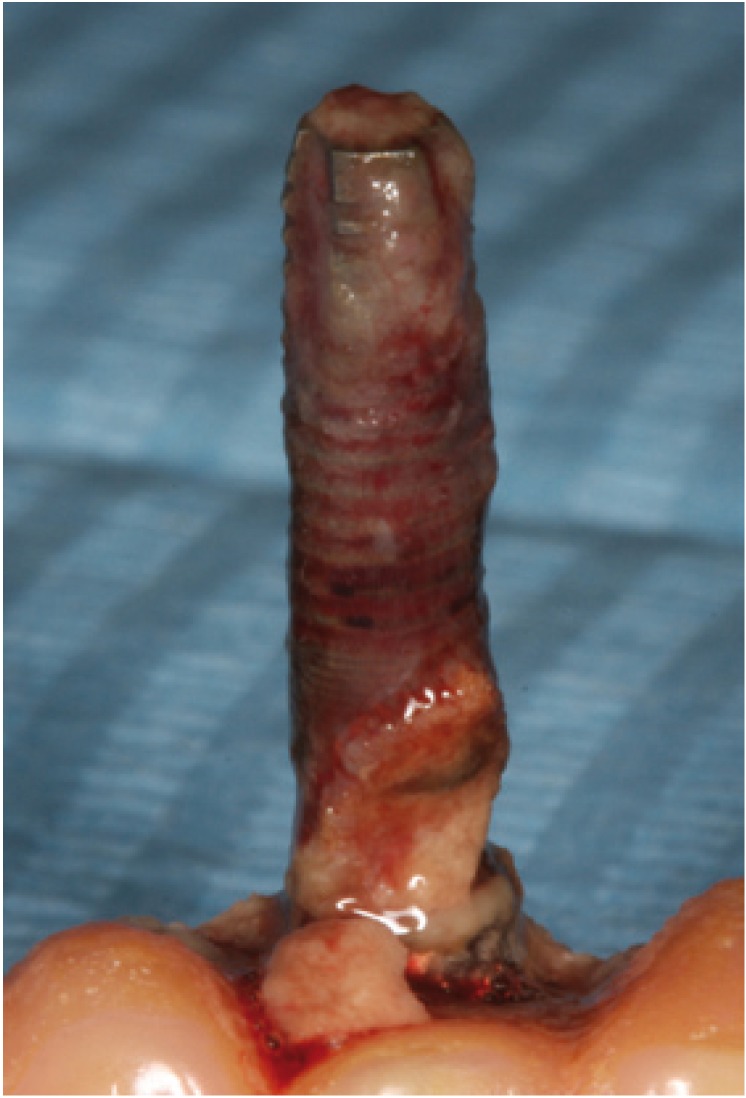
Failed implant in a patient who did not receive supportive periodontal therapy.

**Figure 3:**
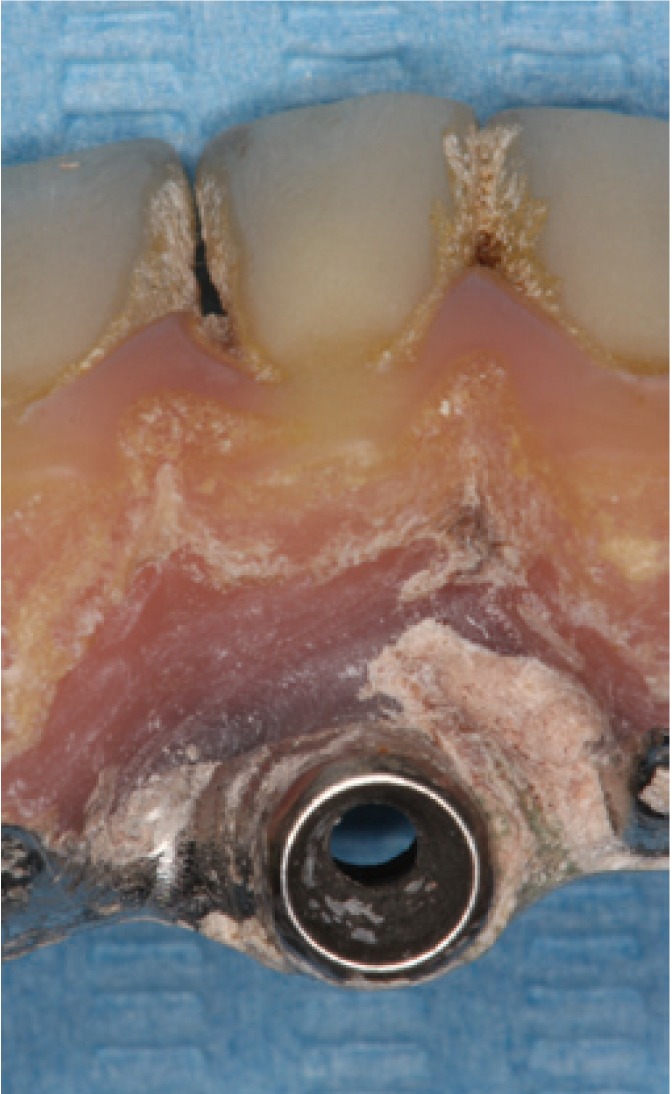
Prosthesis in which a large quantity of retained calculus can be observed in a patient who did not receive maintenance therapy.

One implant was lost during this year (Figs. 2,3), rendering a total survival rate of 99.59% (100% for SPT and 99.18% for No SPT). Six patients (12%) presented typical signs of periimplantitis in at least one fixture, five in the No SPT group (22.7%) and one in the SPT group (3.7%). Furthermore, it was observed that sixteen patients (32%) presented an implant with signs of mucositis, eleven in the No SPT group (50%) and five in the SPT group (18.5%).

The mean plaque index was 39.98% [17-90]. The mean plaque index was 20.34% [17-24] in the SPT group and 59.63% [38-90] in the No SPT group, with a statistically significant correlation with respect to bone loss around their fixtures during the first year (p= 1.59×10–8). ( Table 1).

**Table 1:**
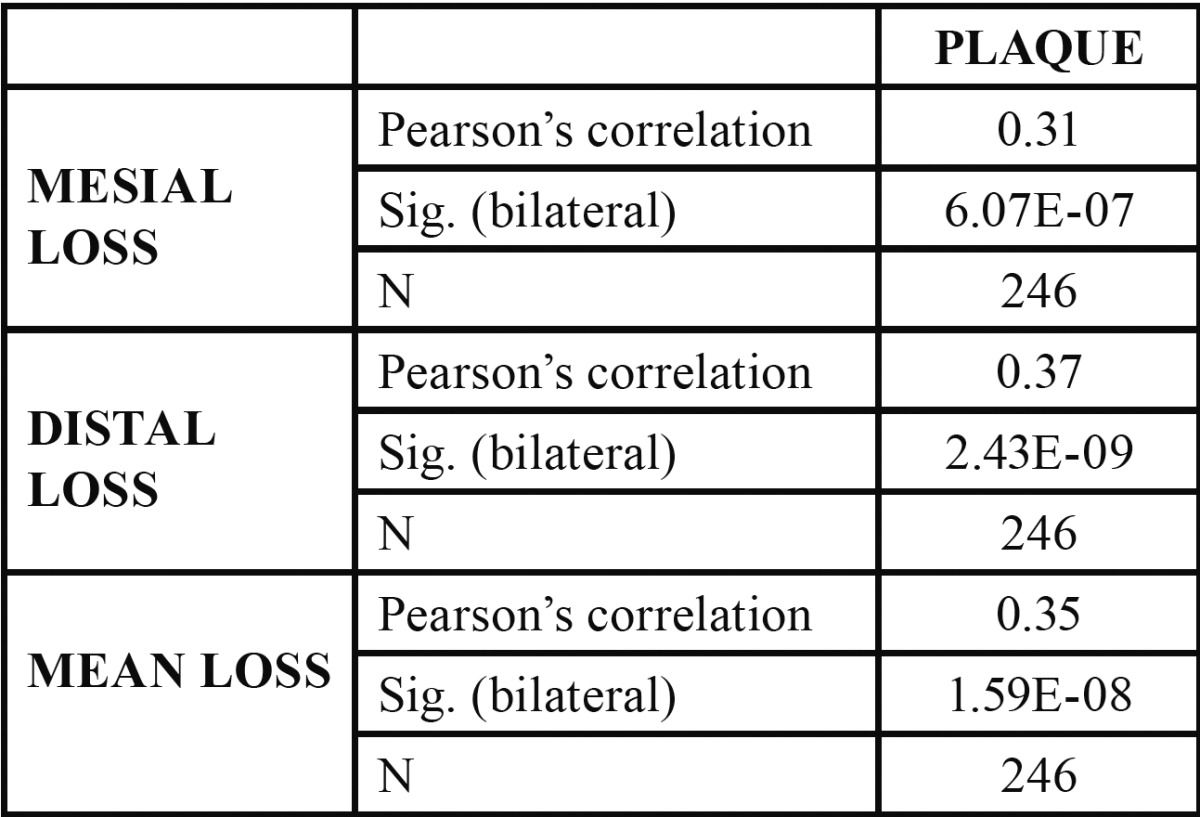
Correlations between bacterial plaque and bone loss (Numerical value from 0 to 1).

Mean bone loss was 0.39 mm (SD 0.71; range [(-0.71)-8.05]), with distal loss (0.45 mm; SD 0.75) being greater than mesial loss (0.33 mm; SD 0.70). In patients who received SPT, mean bone loss was 0.16 mm (SD 0.15; [0-1.24]), while in the No SPT group a loss of 0.62 mm (SD 0.94; range [(-0.71)-8.05]) was observed. This difference was statistically significant (p= 5.15×10–13) ( Table 2). Furthermore, in the SPT group, 95.9% of the implants (118) experienced less bone loss than the mean versus 53.7% (66) of the implants in the No SPT group.

**Table 2:**
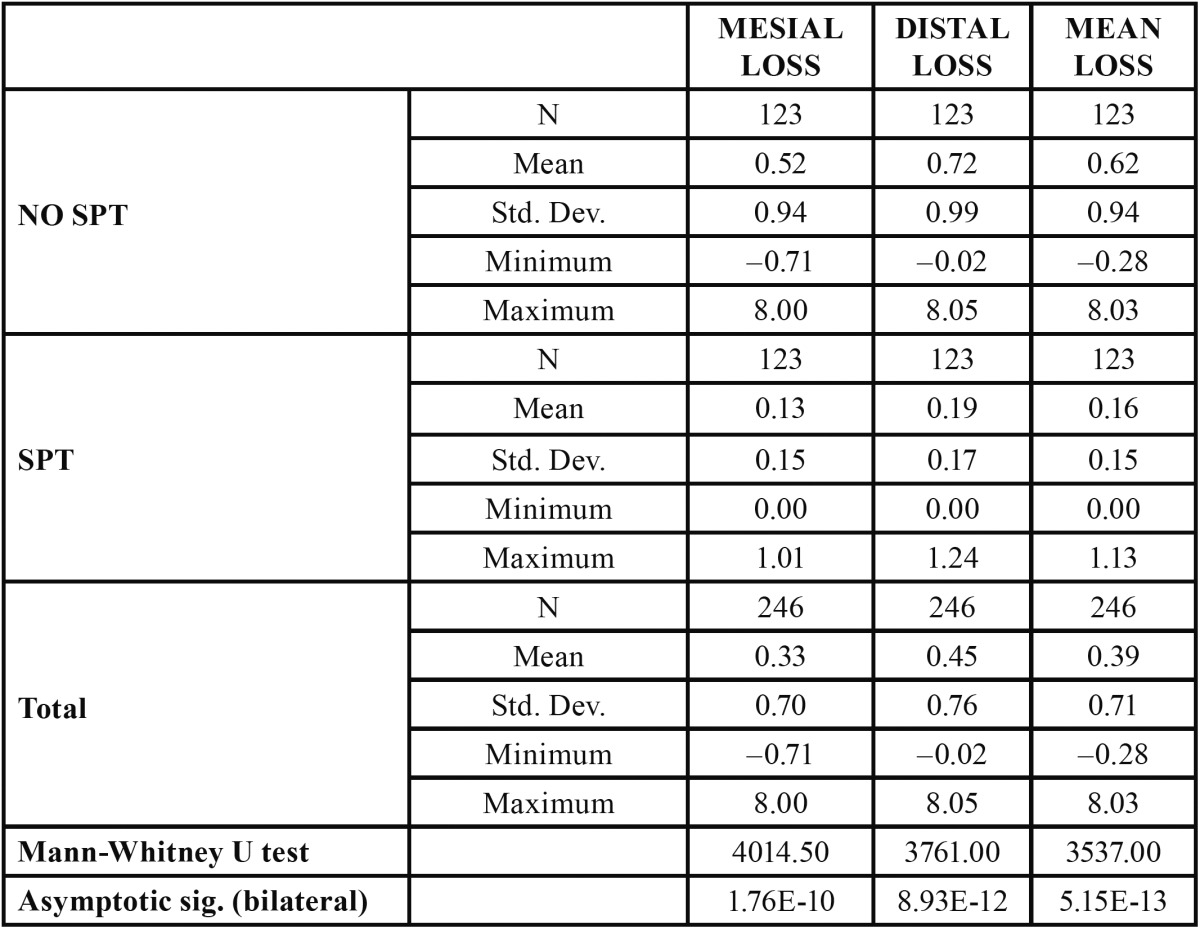
Attachment loss level at 12 months in SPT and No SPT implants (in millimetres).

To assess the influence of periodontal biotype, the Kruskal-Wallis test was applied and significant differences were observed in bone loss (p= 1.31×10–16) in the different groups, although the most evident results were observed when comparing the fixtures placed in patients with a thick biotype in the SPT group (64 implants in 16 subjects), with a mean bone loss of 0.09 mm (SD 0.08; [0-0.45]), to the implants placed in patients with a thin biotype in the No SPT group (73 fixtures placed in 11 patients), with a mean bone loss of 0.78 mm (SD 1.14; [(-0.23)-8.05]) ( Table 3).

**Table 3:**
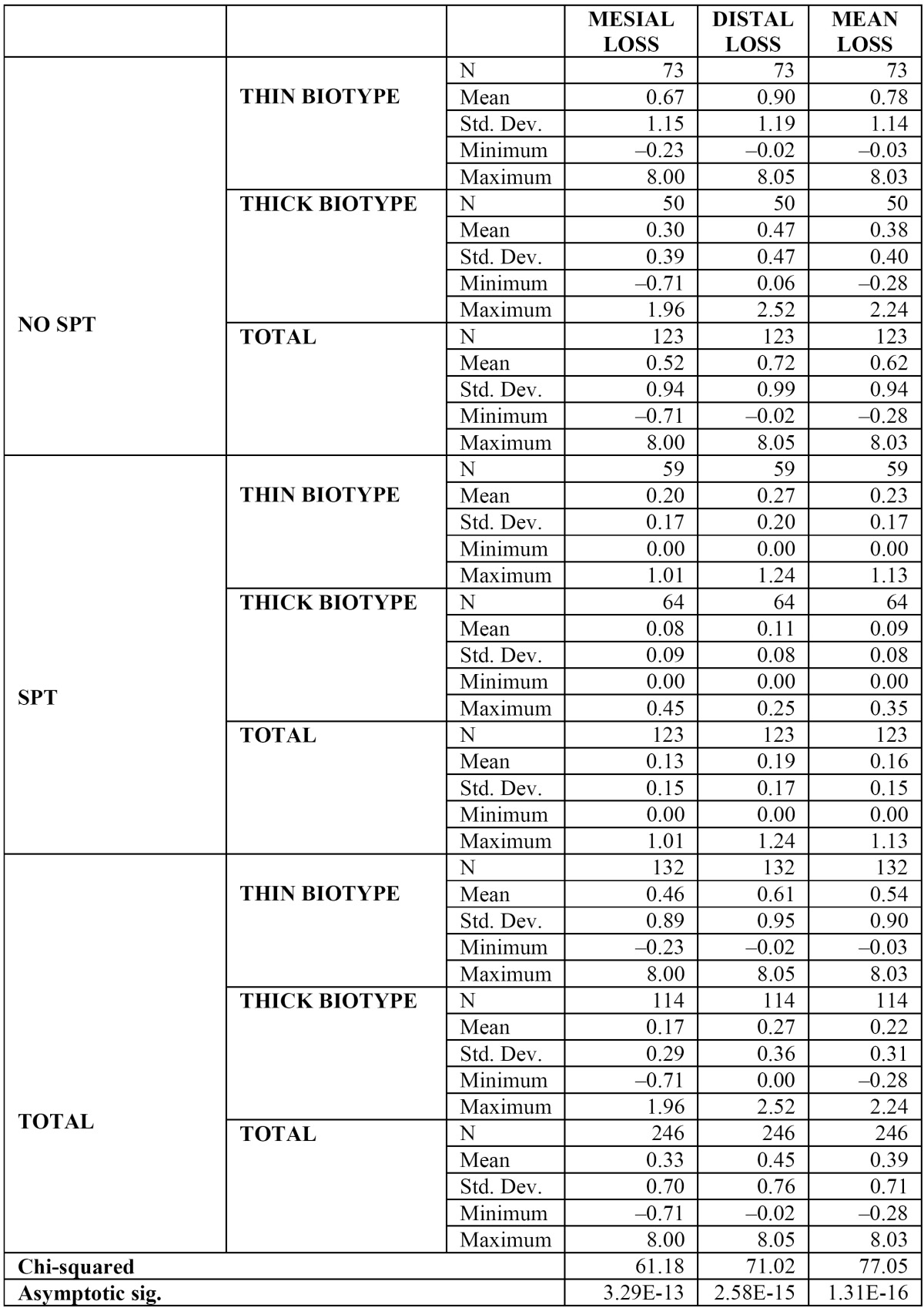
Attachment loss level at 12 months in SPT and No SPT implants and according to periodontal biotype (in millimetres).

Five of the six cases of periimplantitis mentioned above were related to thin biotypes and one to thick biotype. Similarly, of the sixteen cases of mucositis, twelve were related to thin biotypes and four to thick biotypes.

With respect to the bleeding index and probing depth, given the design of some of the prostheses and the difficulty in correctly evaluating these parameters in each and every patient and implant, a correct statistical analysis could not be performed. A 24.2% of the prostheses could not be probed due to their design, being these prostheses related with four of the patients with periimplantitis (66.6%) and thirteen of the patients with mucositis (81.2%).

## Discussion

The studies published on the Astra Tech® implant system in professionally monitored patients with good hygiene report mean bone losses during the first year in function between 0.02 and 0.4 mm (20,21).

Cecchinato et al. (22) studied the bone changes around Astra Tech® implants analysing 115 fixed partial prostheses supported by 324 fixtures in 84 patients. The implant placement protocol used procedures in one and two stages (groups A and B) and mean bone losses during the first year of functional loading of 0.02 and 0.17 mm were observed for the respective groups. The results obtained by Wennstrom et al. (23) with regard to the single fixed prosthesis were similar. A total of 45 fixtures were analysed, with a mean bone loss of 0.02 mm during the first year in function.

For other implant systems and the “periodontally stable” patient type, normal bone loss is considered to be between 0.1 and 1.6 mm during the first years of functional loading (24,25).

Studies published on the long-term evolution of implants placed in patients who have previously been treated for chronic periodontitis do not contraindicate their installation, although studies suggest a lower survival rate and greater number of biological complications (26,27).

The number of fixtures with complications in periodontal patients after loading is in the range of 0-21% (26-29). However, when these patients are included in an SPT programme this range is greatly reduced [0-3.9%] (28,29).

Wennström et al. (30) observed a mean bone reduction of 0.33 mm during the first year in function in 149 Astra Tech® implants placed in 51 patients who had been treated for chronic periodontitis and included in a maintenance protocol.

It is worth mentioning the study by Mengel and Flores-de-Jacoby (29), in which the authors compared the results of 150 implants (Brånemark System Implants® and 3i Implant Innovations®) placed in three patient groups: periodontally healthy patients, patients previously treated for chronic periodontitis and patients treated for generalised aggressive periodontitis. Loss of bone level after one year was 0.58 mm, 0.68 mm and 0.83 mm respectively, increasing at three years to 0.12 mm, 0.18 mm and 0.31 mm for the different groups. This study would suggest that not only is periodontitis a risk factor for attachment loss, but also that the type of periodontitis can have an effect.

In the present study, the survival rate during the first year in function was 99.59% (100% SPT and 99.18% No SPT) and mean bone loss was 0.39 mm, being 0.16 mm in SPT patients and 0.62 mm in the No SPT group. The difference observed between the patients who received SPT and those who did not, can be explained by the correlation observed between plaque index and bone loss (0.35) (Table 1). This value does not establish a causal relationship, but does indicate that there is a greater tendency to bone loss when plaque index is high or that, in the absence of a high plaque index, minor losses will more likely occur.

In this regard, Quirynen et al. (31) emphasize the importance of supportive periodontal therapy in patients with a history of periodontitis and treated with rough surface fixtures.

In this paper, five of the six patients with periimplantitis were subjects who did not attend maintenance and 66.6% of these, together with 81.2% of the patients who presented implants with some sign of mucositis, had prostheses that could not be correctly probed, as demonstrated by Serino and Ström (32).

Periodontal biotype also seems to play a role in patients with implants (15,16). In this study, great differences have been observed between the No SPT patients with thin biotypes, with a mean bone loss of 0.78 mm, and the SPT patients with a thick biotype, in which a mean bone loss of 0.09 mm was observed. These differences would not only be explained by a good plaque control, but also would indicate that the level of bone around a fixture at the moment of its installation is a significant prognostic factor with regard to its evolution over time.

## Conclusion

Within the limitations of this study, e.g. the sample size and the study duration, the results demonstrate the good behaviour of the Astra Tech® implant system during the first year of functional loading in patients with treated periodontal pathology.

Furthermore, it indicates that establishing a regulated maintenance protocol after the active phase of periodontal treatment is essential in order to minimize the risk of marginal bone loss in implants placed in periodontal patients.

It also illustrates the influence that two factors, plaque index and periodontal biotype, have on bone loss after loading the implants, especially when implants placed in thin biotypes without maintenance are compared to fixtures placed in thick biotypes with maintenance.
